# Heliox Improves Carbon Dioxide Removal during Lung Protective Mechanical Ventilation 

**DOI:** 10.1155/2014/954814

**Published:** 2014-12-07

**Authors:** Charlotte J. Beurskens, Daniel Brevoord, Wim K. Lagrand, Walter M. van den Bergh, Margreeth B. Vroom, Benedikt Preckel, Janneke Horn, Nicole P. Juffermans

**Affiliations:** ^1^Laboratory of Experimental Intensive Care and Anaesthesiology (LEICA), Academic Medical Center, University of Amsterdam, Room M0–210, Meibergdreef 9, 1105 AZ Amsterdam, The Netherlands; ^2^Department of Intensive Care, Academic Medical Center, University of Amsterdam, Room M0–210, Meibergdreef 9, 1105 AZ Amsterdam, The Netherlands; ^3^Department of Anaesthesiology, Academic Medical Center, University of Amsterdam, Meibergdreef 9, 1105 AZ Amsterdam, The Netherlands; ^4^Department of Intensive Care, University Medical Center, Hanzeplein 1, 9713 GZ Groningen, The Netherlands

## Abstract

*Introduction*. Helium is a noble gas with low density and increased carbon dioxide (CO_2_) diffusion capacity. This allows lower driving pressures in mechanical ventilation and increased CO_2_ diffusion. We hypothesized that heliox facilitates ventilation in patients during lung-protective mechanical ventilation using low tidal volumes. *Methods*. This is an observational cohort substudy of a single arm intervention study. Twenty-four ICU patients were included, who were admitted after a cardiac arrest and mechanically ventilated for 3 hours with heliox (50% helium; 50% oxygen). A fixed protective ventilation protocol (6 mL/kg) was used, with prospective observation for changes in lung mechanics and gas exchange. Statistics was by Bonferroni post-hoc correction with statistical significance set at *P* < 0.017. *Results*. During heliox ventilation, respiratory rate decreased (25 ± 4 versus 23 ± 5 breaths min^−1^, *P* = 0.010). Minute volume ventilation showed a trend to decrease compared to baseline (11.1 ± 1.9 versus 9.9 ± 2.1 L min^−1^, *P* = 0.026), while reducing PaCO_2_ levels (5.0 ± 0.6 versus 4.5 ± 0.6 kPa, *P* = 0.011) and peak pressures (21.1 ± 3.3 versus 19.8 ± 3.2 cm H_2_O, *P* = 0.024). *Conclusions*. Heliox improved CO_2_ elimination while allowing reduced minute volume ventilation in adult patients during protective mechanical ventilation.

## 1. Introduction

Helium is an inert gas with lower density than air [1], allowing for less turbulent flow through airways and leading to lower airway resistance. As a result, during mechanical ventilation with a helium/oxygen mixture (heliox), lower driving pressures are needed to distribute oxygen to the distal alveoli compared to ventilation with oxygen [2]. Furthermore helium is known for its increased diffusion capacity of carbon dioxide (CO_2_), which in addition might facilitate ventilation. Due to these properties, there may be a rationale to use heliox in patients with severe pulmonary disease with respiratory failure whose protective mechanical ventilation with low tidal volumes is not feasible due to the development of respiratory acidosis, for example, in acute respiratory distress syndrome (ARDS) or chronic obstructive pulmonary disease (COPD). Nowadays, the use of heliox is clinically applied using high frequency ventilation in pediatric patients [3, 4] and in patients with high airway resistance due to severe asthma or COPD, most often in children [5, 6]. Clinical data on adult patients during conventional mechanical ventilation are limited.

The aim of this study was to investigate the effect of heliox on gas exchange as part of a safety and feasibility study on the potential of heliox ventilation to improve neurological outcome after cardiac arrest [7]. We hypothesized that the use of heliox also allows for increased CO_2_ elimination in adults during conventional mechanical ventilation with low tidal volumes.

## 2. Methods

The study was approved by the local medical ethics committee of the Academic Medical Center, University of Amsterdam, The Netherlands (Protocol number NL 30466.018.09), and conducted in concordance with the principles of the declaration of Helsinki and good clinical practice. The study was registered with the Dutch Trial Registry (http://www.trialregister.nl) under NTR2257. From all patients or their legal surrogate written informed consent was obtained. It was a prospective observational cohort substudy of an open label single arm intervention study, performed in the mixed surgical-medical intensive care unit (ICU) of a tertiary referral center in Amsterdam, The Netherlands. From April 2010 to October 2011, consecutive patients admitted to the ICU after cardiopulmonary resuscitation (CPR) because of a witnessed out-of-hospital cardiac arrest were screened for inclusion in the study after informed consent was given by their relatives. Inclusion criteria were return of spontaneous circulation within 30 minutes of arrest and coma on admission. Exclusion criteria were hypoxemia with a need for ventilation with a FiO_2_ higher than 50% or more than 10 cm H_2_O positive end-expiratory pressure (PEEP), pregnancy, severe disability, a neurological disorder, or comorbidity with life expectancy of less than 6 months.

Before the start of heliox ventilation, all patients were ventilated in a pressure-controlled mode, targeting a pH of 7.35–7.45 and PaCO_2_ levels of 4.5–5.5 kPa. Within 5 hours after the cardiac arrest patients received heliox ventilation. During heliox treatment patients were mechanically ventilated in a pressure controlled mode, using a Servo-I ventilator, which was adjusted and calibrated for heliox ventilation. Helium (Linde Gas Therapeutics, Eindhoven, The Netherlands) was mixed with oxygen to achieve a concentration of 50% helium and 50% oxygen. Respiratory settings were modified using a study protocol. Inspiratory pressure and respiratory rate were adjusted to target a tidal volume of 6 mL/kg predicted body weight, a pH of 7.35–7.45, and PaCO_2_ levels of 4.5–5.5 kPa, with an inspiration to expiration (I : E) ratio of 1 : 2. No changes were made to I : E ratio and FiO_2_ and PEEP levels during heliox treatment. After 3 hours, heliox was switched back to oxygen in air and patients were ventilated according to our standard ICU protocol with tidal volumes of 6 mL/kg predicted body weight. All patients were treated with therapeutic hypothermia (32°C–34°C) as part of standard care in patients with decreased consciousness after CPR. Target temperature had been reached by the time heliox ventilation was initiated and was maintained during heliox ventilation. For sedation, propofol and opiates were used. Neuromuscular relaxants were given as a bolus but only during shivering.

Respiratory parameters were measured over time, starting just prior to heliox ventilation (*T* = −1), within 15 minutes after start heliox (*T* = 0), during heliox treatment (*T* = 1–*T* = 3), and until 3 hours after heliox was switched back to oxygen in air (*T* = 4–*T* = 6). After the switch back, again all patients were ventilated in a pressure-controlled mode, targeting a pH of 7.35–7.45 and PaCO_2_ levels of 4.5–5.5 kPa. Dynamic lung compliance was measured during heliox ventilation (*T* = 1–*T* = 3), as this was a read-out at the Servo-I ventilator only. Resistance was calculated by dividing the pressure difference by airflow per minute. Arterial blood gas analysis was determined hourly (Alpha stat, RAPIDLab 1200, Siemens, Deerfield, USA).

### 2.1. Statistical Analysis

Data are expressed by means and the standard error of the mean (SEM) in the figures. Time points within the same subjects were compared using paired *t*-test or Wilcoxon signed rank test, depending on distribution of the data. A total of three comparisons were made between several time points (*T* = −1 versus *T* = 0; *T* = 0 versus *T* = 3; *T* = 3 versus *T* = 6). Using Bonferroni post-hoc correction, statistical significance was set at *P* < 0.017.

## 3. Results

106 patients were screened, of whom 29 were eligible. Of these, informed consent was refused in 4 cases. Of 25 included patients, heliox was discontinued within 15 minutes in one patient due to hypoxemia, requiring a PEEP level above 10 cm H_2_O. This patient was excluded from further analyses. In the remaining 24 patients, PEEP requirements were 5–10 cm H_2_O and 40–50% FiO_2_. Of these, 83% were male with a mean age of 65 ± 12 years; no acute infections were present at start of the study; 1 patient suffered from COPD; no other lung pathology was reported. During the study protocol, no changes in hemodynamics were observed.

**Figure 1:**
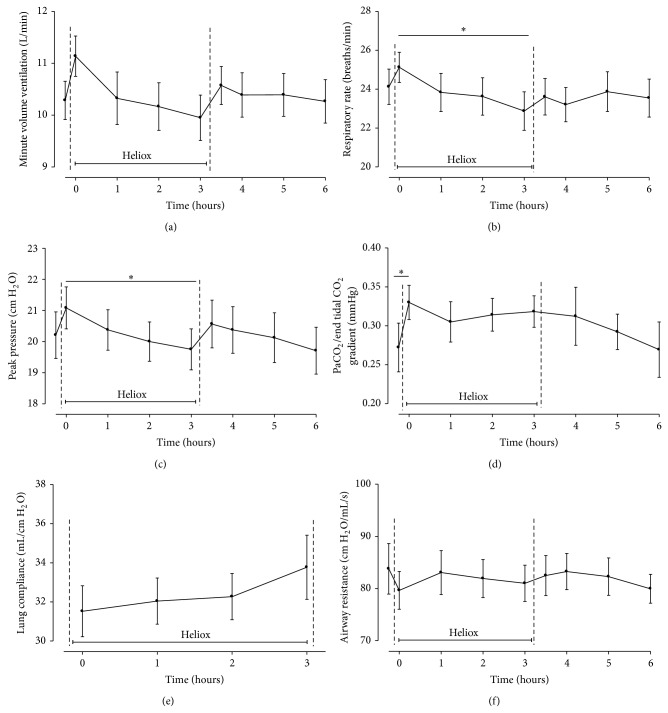
Respiratory parameters during heliox ventilation for 3 hours (*T* = 0 to *T* = 3) and after switch to normal oxygen in air mixture (*T*3 to *T*6). Measurements started prior to heliox administration (*T* = −1). Data are means ± SEM. (a) Minute volume ventilation (L min^−1^); (b) respiratory rate (breaths min^−1^); (c) peak pressure (cm H_2_O); (d) PaCO_2_/end tidal CO_2_ gradient (mmHg); (e) airway resistance (cm H_2_O mL^−1^ sec^−1^); and (f) lung compliance (mL cm^−1^ H_2_O). ∗: *P* < 0.02.

Due to the switch of ventilation gas mixture from oxygen (*T* = −1) to heliox (*T* = 0), respiratory settings needed adjustment according to the study protocol with limited tidal volume ventilation. Minute volume ventilation slightly rose after switching from oxygen to heliox, but no significant difference was found between before and right after the start of heliox ventilation (Figure 1). Thereafter, during heliox ventilation, respiratory rates were adjusted to targeted pH and PaCO_2_ levels, in accordance with the study protocol. This resulted in a significant decrease in respiratory rate and tended to decrease minute volume ventilation (Figure 1). After discontinuation of helium these parameters did not change. Tidal volumes remained stable at 6 mL/kg according to study and standard ICU protocol and did not change over time (data not shown). Peak pressures tended to decrease, albeit in a nonsignificant manner (Figure 1). Airway resistance and dynamic lung compliance by the ventilator did not change during heliox ventilation (Figure 1).

**Figure 2:**
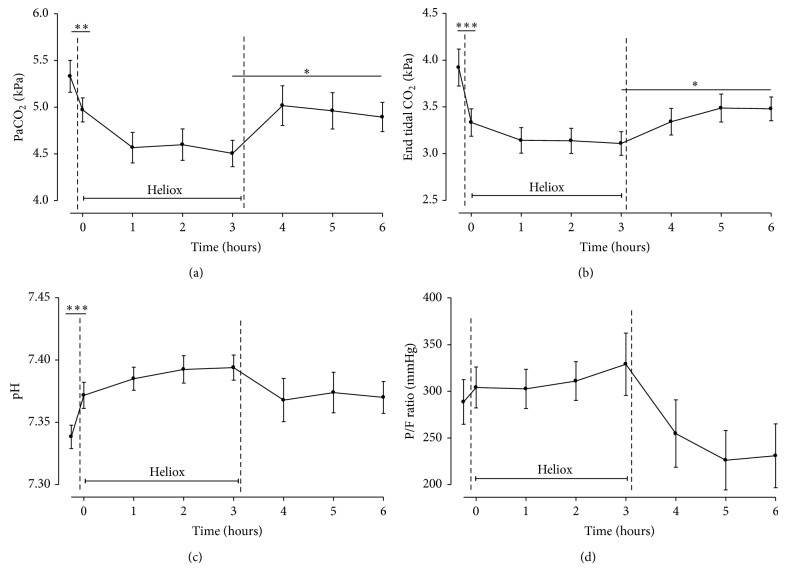
Gas exchange during ventilation with heliox for 3 hours (*T* = 0 to *T* = 3). Measurements started just prior to heliox administration (*T* = −1) until 3 hours after heliox discontinuation (*T* = 3 to *T* = 6). Data are means ± SEM. (a) PaCO_2_ (kPa); (b) end tidal CO_2_ measurements (kPa); (c) pH measured hourly; and (d) PaO_2_/Fi O_2_ ratio (mmHg). ∗: *P* < 0.02; ∗∗: *P* < 0.01; ∗∗∗: *P* < 0.001.

Switch from oxygen in air to heliox ventilation resulted in a rapid decrease in PaCO_2_ levels, which increased again at discontinuation of heliox (Figure 2). PaCO_2_ levels showed no changes during the 3 hours of heliox ventilation (Figure 2). Also, an increase in pH to 7.37 was seen shortly after the application of heliox ventilation (Figure 2). In the course of heliox ventilation, pH tended to increase further but showed no change after discontinuation of heliox. Oxygenation was not altered significantly after the start of heliox or after switching back to oxygen (Figure 2). Applied FiO_2_ levels remained between 40 and 50%, whereas PaO_2_ levels tended to decrease.

## 4. Discussion

In adult patients ventilated with protective mechanical ventilation strategy according to current ventilation guidelines [8], the use of heliox improved ventilation, by allowing lower minute volume ventilation while PaCO_2_ levels decreased.

The use of heliox ventilation has been mostly investigated in respiratory conditions such as upper-airway obstruction, asthma, bronchiolitis, and croup. Results indicate that heliox improves gas exchange and reduces work of breathing [4–6]. Most of the studies were performed in the pediatric population. In this study we focused on adult patients. Cardiac arrest patients are obviously not the patients who are expected to benefit most from lowering minute volume ventilation, because these patients do not have obstructed airflow. Nevertheless this population was studied, since the feasibility study investigating neuroprotective properties of heliox [7] enabled us to investigate the response to heliox ventilation in adult patients ventilated with pressure controlled ventilation modes and currently recommended protective settings. The reduction of respiratory rate with the concomitant decrease of peak pressures during heliox ventilation is promising result. Given that changes were small, it can, however, be questioned whether these results have clinical relevance. Effects of heliox may have been mild because baseline resistance as well as compliance in these patients without lung injury was not severely hampered. It remains to be determined whether heliox is beneficial in patients with respiratory failure in whom protective ventilation is hampered by the development of respiratory acidosis.

Our study has several limitations. As this study was a secondary analysis of a safety and feasibility study on the use of heliox in cardiac arrest patients, the number of patients was not primarily powered to investigate the effects of heliox on ventilation. This may explain observed trends but absence of statistical significance. Also, no control group was present. Thereby, the influence of time on findings is unknown. However, our data clearly show an increased CO_2_ removal and improved ventilation, starting immediately after start of heliox ventilation. Long-term effects could not be studied as heliox ventilation was limited to 3 hours. Another limitation may be that all patients received therapeutic hypothermia, which is known to decrease PaCO_2_ levels [9]. However, throughout the whole study period, temperatures were in the range of therapeutic induced hypothermia. Thereby, observed effects could not be due to hypothermia.

## 5. Conclusions

Heliox ventilation improved CO_2_ elimination and allowed for, though nonsignificant, decrease in minute volume ventilation, in a selected group of patients ventilated in a pressure controlled mode according to the guidelines of the ARDS network [8]. Results may generate new hypotheses for future research considering heliox as a therapeutic possibility in patients whose protective mechanical ventilation is hampered by the development of respiratory acidosis.
